# Supplementary material for: Binding Specificity and Oligomerization of TSWV N Protein in the Western Flower Thrips, Frankliniella occidentalis
Source: Viruses. 2025 Jun 7;17(6):826. doi: 10.3390/v17060826 (PMC12197737; doi:10.3390/v17060826)

Supplementary information:

Table S1. Primers and probes used in this study.

Table S2. GenBank accession numbers of the N protein genes of different orthotospoviruses.

Figure S1. Thermal shifting assays for controls of four different nucleic acids without TSWV-N protein.

Table S1. Primers and probes used in this study.

Genes	Sequence (5'-3')	Uses	Annealing Temperature (^{0}C)	Expected Size (bp)
Fo-Gn	GTCAAGTCCGTCCCCCAGTA<br>GTCGAACTCCTGAACCTGGC	RT-PCR	55.0	312
T7+ Fo-Gn	TAATACGACTCACTATAGGGAG GTCAAGTCCGTCCCCCAGTA<br>TAATACGACTCACTATAGGGAG GTCGAACTCCTGAACCTGGC	RNAi	55.0	358
TSWV-N	ATGTCTAAGGTTAAGCTCACTAAGGAA<br>TTAAGCAAGTTCTGCAAGTATTGCCTG	RT-PCR<br>RT-qPCR	55.0	777
TSWV-NSs	ATGTCTTCAAGTGTTTATGAGT<br>TTTTGATCCTGAAGCATACGCT	RT-PCR	55.0	1403
T7+TSWV-NSs	TAATACGACTCACTATAGGGAGA ATGTCTTCAAGTGTTTATGAGT<br>TAATACGACTCACTATAGGGAGA TTTTGATCCTGAAGCATACGCT	RNAi	55.0	1449
Elongation Factor 1	TCAAGGAACTGCGTCGTGGAT<br>ACAGGGGTGTAGCCGTTAGAG	RT-qPCR	55.0	160
TSWV-N	FAM-TTTTTAAGCAAGTTCTGCAAGTATTGC CTG<br>FAM-AAACAGGCAATACTTGCAGAACTTGC TTAA	FISH	-	-
TSWV-NSm	Rhodamine-AAAATGGATACATCCAAAGGAAA GATACTG<br>Rhodamine – TTTGTTGCTCAGTTATCTTTTGA TGAGATA	FISH	-	-
TSWV-RdRp	FAM-AAAATGAACATCCAGAAAATACAAAA ATTA<br>FAM- TTTCACAACAACAGGCTTGGCAT	FISH	-	-

Table S2. GenBank accession numbers of the N protein genes of different orthotospoviruses.

Abbreviation	Orthotospovirus Name	Accession Number
TSWV	Tomoato spotted wil virus	AWV56668.1
ANSV	Alstroemeria necrotic streak virus	AWV56668.1
CCV	Capsicum chlorosis virus	AAX54506.1
GRSV	Groundnut ringspot virus	AYA95027.1
HCSV	Hippeastrum chlorotic spot virus	AUN35174.1
INSV	Impatiens necrotic spot virus	AXX39061.1
IYSV	Iris yellow spot virus	BAG06877.1
MYSV	Melon yellow spot virus	BCO07624.1
SVNV	Soybean vein necrosis virus	ADX42520.1
TCSV	Tomato chlorotic spot virus	QBC75816.1
TYFRV	Tomato yellow fruit ring virus	AGU99115.1
TYRV	Tomato yellow ring virus	ABF59486.1
TZSV	Tomato zonate spot virus	WAB75057.1
WBNV	Watermelon bud necrosis virus	ABY79095.1
WSNV	Watermelon silver mottle orthotospovirus	CAA86555.1
ZLCV	Zucchini lethal chlorosis virus	AAF04198.1

Figure S1. Thermal shifting assays for controls of four different nucleic acids without TSWV-N protein.

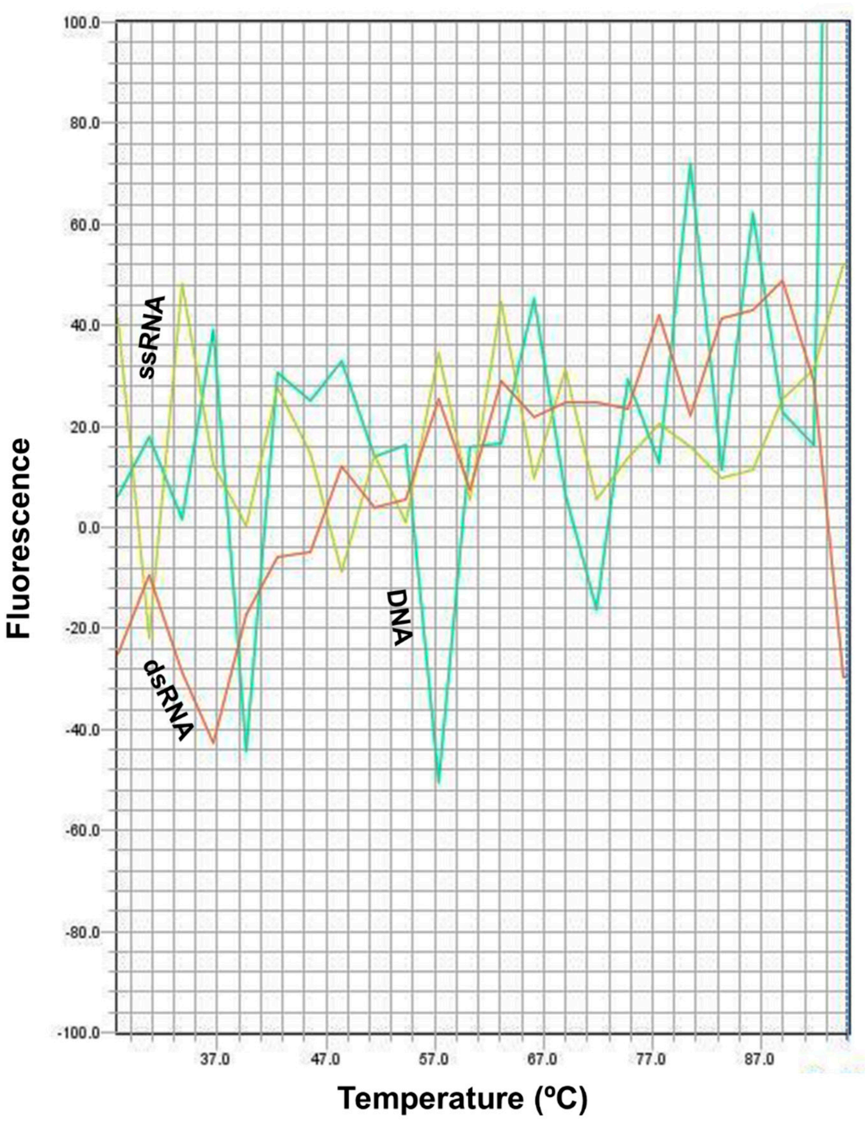

Supplement: Supplementary file 1 [file viruses-17-00826-s001.zip › viruses-3629714-supplementary.pdf]
